# IL-6 ameliorates acute lung injury in influenza virus infection

**DOI:** 10.1038/srep43829

**Published:** 2017-03-06

**Authors:** Mei-Lin Yang, Chung-Teng Wang, Shiu-Ju Yang, Chia-Hsing Leu, Shun-Hua Chen, Chao-Liang Wu, Ai-Li Shiau

**Affiliations:** 1 Department of Microbiology and Immunology, College of Medicine, National Cheng Kung University, Tainan, Taiwan; 2 Department of Biochemistry and Molecular Biology, College of Medicine, National Cheng Kung University, Tainan, Taiwan

## Abstract

Interleukin 6 (IL-6) is involved in innate and adaptive immune responses to defend against pathogens. It also participates in the process of influenza infection by affecting viral clearance and immune cell responses. However, whether IL-6 impacts lung repair in influenza pathogenesis remains unclear. Here, we studied the role of IL-6 in acute influenza infection in mice. IL-6-deficient mice infected with influenza virus exhibited higher lethality, lost more body weight and had higher fibroblast accumulation and lower extracellular matrix (ECM) turnover in the lung than their wild-type counterparts. Deficiency in IL-6 enhanced proliferation, migration and survival of lung fibroblasts, as well as increased virus-induced apoptosis of lung epithelial cells. IL-6-deficient lung fibroblasts produced elevated levels of TGF-β, which may contribute to their survival. Furthermore, macrophage recruitment to the lung and phagocytic activities of macrophages during influenza infection were reduced in IL-6-deficient mice. Collectively, our results indicate that IL-6 is crucial for lung repair after influenza-induced lung injury through reducing fibroblast accumulation, promoting epithelial cell survival, increasing macrophage recruitment to the lung and enhancing phagocytosis of viruses by macrophages. This study suggests that IL-6 may be exploited for lung repair during influenza infection.

Influenza causes worldwide yearly epidemics and results in significant morbidity and mortality. Influenza-associated lung injury is attributable to the virus and the bystander problems evoked through the imbalance of inflammatory cells, fibroblasts and epithelial cells in the lung. Previous studies have shown that patients or fatal cases with novel swine-origin influenza A (H1N1) virus infection had pulmonary inflammation, and some displayed fibrosis progression^1^,^2^ and acute respiratory distress syndrome (ARDS)^3^, suggesting appropriate lung repair is crucial in influenza.

A number of growth factors, such as transforming growth factor (TGF)-β, interleukin (IL)-22 and IL-27, are involved in lung injury, repair and regeneration during influenza infection^4^,^5^,^6^,^7^. TGF-β primarily secreted by fibroblasts, epithelial cells and macrophages is the most important factor. It promotes fibroblast proliferation, resistance to apoptosis and collagen production, as well as induces epithelial-mesenchymal transition (EMT)^8^. TGF-β is also a key mediator for acute lung injury (ALI) and is elevated in the lung fluid of patients with ALI/ARDS^9^,^10^. Moreover, it promotes internalization of the epithelial sodium channel (ENaC), thereby retaining lung fluids and resulting in edema^11^. Influenza infection induces TGF-β production, leading to apoptosis of epithelial cells^12^,^13^. Infection with influenza virus also stimulates Toll-like receptor 3 (TLR3), which activates TGF-β and causes epithelial cell death through αvβ6 integrin^4^. However, the mechanism by which TGF-β impacts lung repair process in influenza remains unclear.

Although the role of IL-6 in influenza pathogenesis has been documented, to date no studies have investigated its role in modulating lung repair responses necessary for recovery from influenza. IL-6 exerts diverse functions in regulating innate and adaptive immune systems to defend against influenza infection^14^,^15^,^16^,^17^. In severe patients with H1N1 influenza, increased levels of several cytokines including IL-6 were detected, which were the hallmarks for disease severity^18^,^19^. Nevertheless, IL-6 knockout mice have similar morbidity and mortality rates to wild-type (WT) mice after infection with highly pathogenic H5N1 influenza virus^20^,^21^. It is still obscure how IL-6 controls influenza-induced pneumonia, the subsequent lung fibrosis and regeneration of epithelial cells from severe injury after influenza infection.

In the present study, with the use of a mouse model of ALI after influenza, we elucidate the functions of IL-6 in regulating the balance among fibroblasts, macrophages and epithelial cells by stabilizing extracellular matrix (ECM) turnover and in recovery from lung injury probably through suppressing TGF-β production. Moreover, IL-6 prevents virus-induced apoptosis of lung epithelial cells and enhances phagocytosis of viruses by macrophages. Our findings indicate that IL-6 increases fibroblast apoptosis, macrophage phagocytic activity and epithelial cell survival. We show that IL-6 not only acts as an immune regulator to defend against influenza, but also plays an important role in balancing lung environment. Furthermore, this study sheds some lights on the processes of lung injury and repair during influenza infection.

## Results

### Mice lacking IL-6 are more susceptible to lethal infection with influenza virus

**Figure 1:**
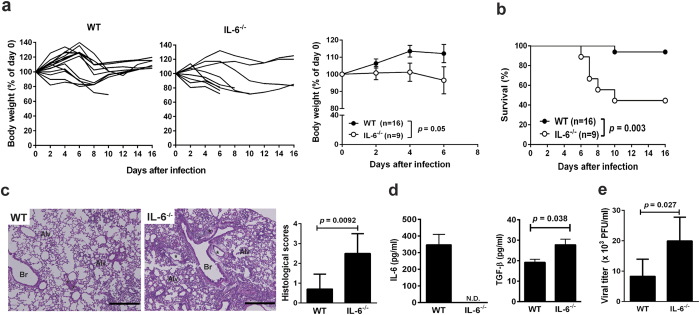
IL-6 deficiency increases mouse lethality and promotes lung injury. IL-6^−/−^ and WT mice were intranasally inoculated with 10^5^ PFU of IAV at day 0. (**a**) Changes in body weights in the individual WT (left) and IL-6^−/−^ (middle) mice, and mean changes in body weights from day 0 through day 6 while all mice were still alive (right). Body weights were recorded and expressed as a percentage of pre-infection (day 0) body weight. (**b**) Kaplan-Meier survival curves. The data in a and b were pooled from two independent experiments. (**c**) Representative H&E-stained lung sections collected at day 6 p.i. (original magnification ×200, scale bar = 200 μm) (left and middle). Br and Alv indicate bronchiole and alveolus, respectively, and *indicates microvessel. Lung histologic scores based on the severity of inflammation and tissue damage (*n* = 5 for WT, *n* = 6 for IL-6^−/−^) (right). (**d**) Levels of IL-6 and TGF-β in the BAL fluid at day 6 p.i. determined by ELISA (*n* = 4 for WT, *n* = 5 for IL-6^−/−^). N.D., not detectable. (**e**) Viral loads in the BAL fluid at day 6 p.i. determined by the plaque assay (*n* = 4).

To study the role of endogenous IL-6 in host defense against influenza, we compared the body weight change and survival curves, as well as histological and immunological changes between IL-6-deficient (IL-6^−/−^) and WT C57BL/6 mice after intranasal infection of influenza A/WSN/33 (H1N1) virus (IAV). As shown in Fig. 1a, four out of nine IL-6^−/−^ mice continued to lose weight and died between 6 and 10 days after infection (middle panel), whereas only one out of 16 WT mice lost weight without weight gain and died at day 10 post-infection (p.i.) (left panel). All of the mice that survived for more than 10 days recovered and survived for at least 16 days. Analysis of the entire body weight curves of the infected mice from day 0 through day 6 while all the mice were still alive reveals that IL-6^−/−^ mice lost more weight over time on average than WT mice (right panel). Figure 1b shows that deficiency in IL-6 increased the mortality and reduced the survival time in mice after IAV infection. As shown in Fig. 1c, histological examination of the lungs collected at day 6 p.i. revealed that IL-6^−/−^ mice had higher levels of mononuclear cell accumulation (middle panel) and higher histologic scores (right panel) than WT mice (left and right panels). In the broncoalveolar lavage (BAL) fluid, IL-6 contents were undetectable (left panel) as expected, whereas levels of TGF-β were higher (right panel) in IL-6^−/−^ mice than in WT mice (Fig. 1d). Furthermore, IL-6^−/−^ mice had significantly higher viral loads than WT mice in the BAL fluid at day 7 p.i. (Fig. 1e). Taken together, these results suggest that IL-6 may be involved in the protection against influenza.

### IL-6^−/−^ mice exhibit increased fibroblast accumulation and reduced ECM turnover in the lung following influenza infection

**Figure 2:**
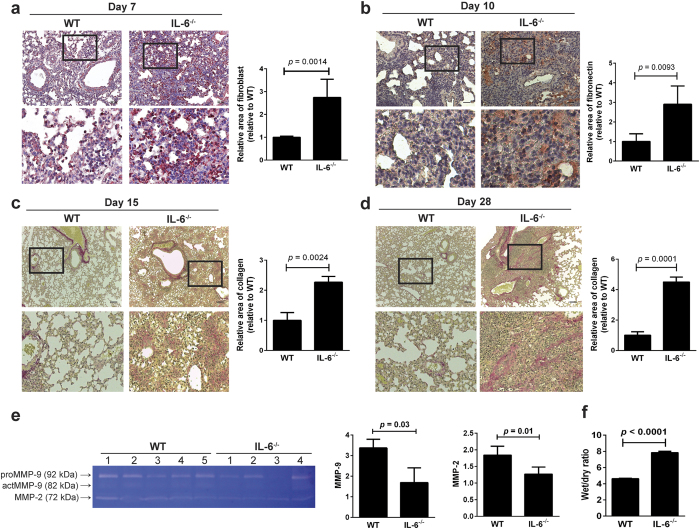
IL-6 deficiency reduces resolution from severe lung injury during influenza infection. IL-6^−/−^ and WT mice were intranasally inoculated with 10^5^ PFU of IAV at day 0. (**a**) Immunohistochemical detection (left) and quantitation (right) of fibroblasts in the lung of mice at day 7 p.i. by monoclonal antibody specific for SFP-1 (original magnification ×200, scale bar = 50 μm). (**b**) Immunohistochemical detection (left) and quantitation (right) of fibronectin in the lung of mice at day 10 p.i. (original magnification ×200, scale bar = 50 μm). (**c**,**d**) Detection of collagen in the lung at day 15 (**c**) and day 28 (**d**) p.i. by picrosirius red staining (original magnification ×100, scale bar = 100 μm). Positively stained areas in a-d were quantified by an image analysis software (*n* = 3 for WT and *n* = 4 for IL-6^−/−^ in (**a**); *n* = 4 in (**b**); *n* = 3 in (**c**); *n* = 3 in (**d**). The boxed areas are magnified below each panel. (**e**) Detection of MMP-2 and -9 in the BAL fluid at day 6 p.i. by gelatin zymography (left). The bands representing MMP-9 (middle) and MMP-2 (right) were quantified by densitometric analysis (*n* = 5 for WT, *n* = 4 for IL-6^−/−^). (**f**) Wet/dry ratios of the lungs at day 7 p.i. (*n* = 3).

Pneumonia is a common complication of influenza with diffuse alveolar damage, fibroblast proliferation and inflammatory cell infiltration^22^. In some cases, pneumonia finally causes fibrosis accompanied with collagen or ECM deposition and EMT. We therefore compared the levels of fibroblasts, fibronectin, collagen, MMP-9 and MMP-2 in the lungs of IL-6^−/−^ and WT mice infected with IAV. IL-6^−/−^ mice had higher amounts of fibroblasts at day 7 p.i. (Fig. 2a) and expressed higher levels of fibronectin at day 10 p.i. (Fig. 2b) than WT mice. However, the amount of fibronectin expressed in lung fibroblasts did not significantly differ between IL-6^−/−^ and WT mice, as examined by immunofluorescence staining (Supplementary Figure S1a). TGF-β can induce α-smooth muscle actin (α-SMA) expression, which is relevant to fibrosis formation. Given elevated expression of TGF-β in the BAL fluid of the infected IL-6^−/−^ mice (Fig. 1d), we further detected the typical EMT marker α-SMA to assess the potential involvement of IL-6 in the regulation of EMT during influenza infection. We found that expression of α-SMA was similar between WT and IL-6^−/−^ lungs exhibiting either minor or severe fibroblast accumulation (Supplementary Figure S1b). Moreover, contents of lung fibroblasts did not differ between IL-6^−/−^ and WT mice (Supplementary Figure S1c). To determine whether fibrosis occurred in the mice at a later stage of influenza, collagen deposition in the lung was assessed by picrosirius red staining. IL-6^−/−^ mice expressed higher levels of collagen in the lung than WT mice detected at day 15 (Fig. 2c) and day 28 (Fig. 2d) p.i. Matrix metalloproteinases (MMPs) represent a group of enzymes involved in the degradation of ECM components. We further detected MMP-9 and MMP-2 in the BAL fluid by gelatin zymography. IL-6^−/−^ mice expressed lower levels of MMP-9 and MMP-2 compared to WT mice (Fig. 2e), suggesting that deficiency in IL-6 may disturb the degradation and turnover of ECM. Furthermore, the wet-to-dry (wet/dry) ratios of the lungs increased by approximately two-fold in IL-6^−/−^ mice compared to WT mice (Fig. 2f), suggesting that deficiency in IL-6 may lead to severe edema and lung injury. Collectively, these results implicate a protective role for IL-6 in influenza infection by reducing fibroblast accumulation and enhancing ECM turnover in the lung.

### IL-6 deficiency enhances proliferation, migration and survival of lung fibroblasts, as well as increases their production of TGF-β

**Figure 3:**
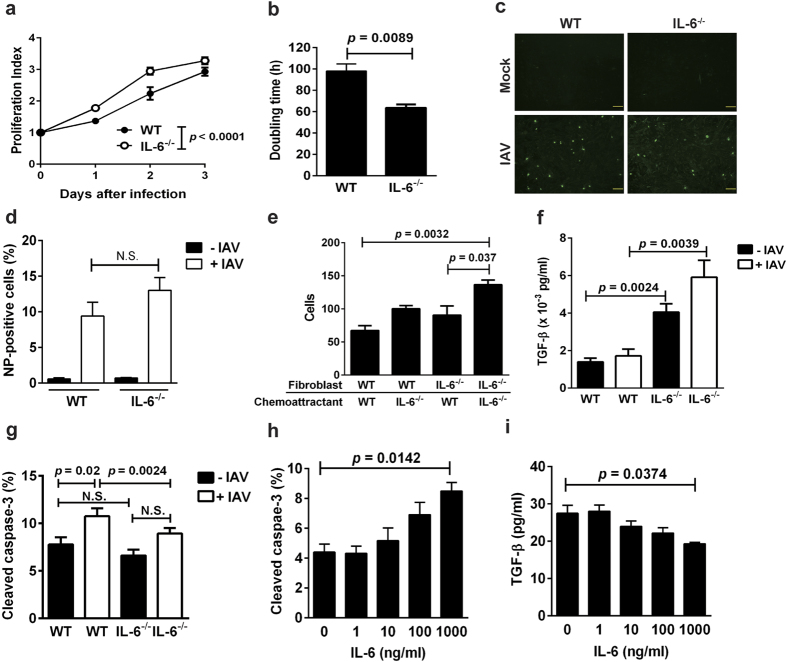
IL-6 deficiency enhances proliferation, migration and survival of lung fibroblasts, as well as increases their production of TGF-β upon influenza infection. Primary lung fibroblasts from WT and IL-6^−/−^ mice were collected and cultured. (**a**) Proliferation rate and (**b**) doubling time of the fibroblasts were calculated with the Celigo cytometry (*n* = 3). (**c**) Immunohistochemical detection and (**d**) quantitation of viral NP in lung fibroblasts of IL-6^−/−^ and WT mice infected with IAV at an MOI of 1 or mock-infected for 24 h (*n* = 4). (**e**) Migratory capability of lung fibroblasts of WT and IL-6^−/−^ mice, as determined by the Boyden chamber assay. The conditioned medium collected from WT and IL-6^−/−^ fibroblasts that had been infected with IAV served as the chemoattractant. The number of migrating cells was the average of the cells counted in three randomly selected fields in each well (*n* = 3). (**f**) Levels of TGF-β in the medium of WT and IL-6^−/−^ fibroblasts with or without IAV infection, as determined by ELISA. Values represent the ratio of the TGF-β content to the total cell number (*n* = 3). (**g**) Percentages of apoptotic fibroblasts from WT and IL-6^−/−^ mice infected with IAV at an MOI of 1 or mock-infected for 24 h (*n* = 3). (**h**) Percentages of apoptotic IL-6^−/−^ fibroblasts infected with IAV at an MOI of 1 in the presence of different concentrations of IL-6 for 24 h. Apoptosis was determined by immunofluorescence staining with anti-cleaved caspase-3 antibody and quantified by the Celigo cytometry (*n* = 3). (**i**) Levels of TGF-β in the medium collected from h, as determined by ELISA (*n* = 3). N.S., non-significant.

To further dissect the effect of IL-6 on fibroblast functions, fibroblasts were isolated from the lungs of IL-6^−/−^ and WT mice and cultured for 72 h. Cell numbers were counted every 24 h using the Celigo cytometer (Cyntellect, San Diego, CA). Increased cell proliferation (Fig. 3a) and decreased doubling time (Fig. 3b) were noted in IL-6^−/−^ fibroblasts compared with those in WT cells. Notably, IL-6^−/−^ and WT lung fibroblasts were equally susceptible to infection by IAV, as assessed by detection of viral nucleoprotein (NP) (Fig. 3c) and quantification of its expression (Fig. 3d). To determine the migratory capability of lung fibroblasts, we used the conditioned medium from IL-6^−/−^ or WT fibroblasts that had been infected with IAV as the chemoattractant. As shown in Fig. 3e, fibroblasts from IL-6^−/−^ mice had a higher migratory capability than those from WT mice in response to the conditioned medium of either WT or IL-6^−/−^ fibroblasts infected with IAV, as determined by the Boyden chamber assay. Notably, IL-6^−/−^ fibroblasts in response to the conditioned medium from IL-6^−/−^ fibroblasts had the highest migratory ability among the four treatment conditions. These results indicate that IL-6^−/−^ lung fibroblasts were more prone to be stimulated to migrate following influenza infection, and that the infected IL-6^−/−^ fibroblasts secreted more chemoattractant proteins capable of stimulating fibroblast migration, as compared with their WT counterparts. Given that IAV-infected IL-6^−/−^ mice produced higher levels of TGF-β in the BAL fluid (Fig. 1d) and their lung fibroblasts displayed higher migratory capability *in vitro* (Fig. 3e) compared with their WT counterparts, we further examined TGF-β levels in the supernatants of IL-6^−/−^ and WT fibroblasts with or without infection with IAV. Figure 3f shows that uninfected IL-6^−/−^ fibroblasts secreted higher levels of TGF-β than their WT counterparts. Notably, levels of TGF-β were further increased when IL-6^−/−^ fibroblasts were infected with IAV, whereas their contents remained similar in WT fibroblasts regardless of viral infection. These results suggest that deficiency in IL-6 may lead to increases in the migratory capability of lung fibroblasts probably through the elevated production of TGF-β.

Fibroblasts from patients with idiopathic pulmonary fibrosis are more active and resistant to apoptosis^23^. To further study whether IL-6 regulated fibroblast survival, we infected lung fibroblasts with IAV and measured the apoptosis of fibroblasts by detection of the cleaved caspase-3. Figure 3g shows that there was a slight reduction in the percentage of cleaved caspase-3 in the infected IL-6^−/−^ fibroblasts in comparison to their WT counterparts. However, in the absence of viral infection, the levels of cleaved caspase-3 were not significantly different between IL-6^−/−^ and WT fibroblasts. To evaluate the importance of IL-6 in apoptosis, we treated IL-6^−/−^ fibroblasts with different doses of recombinant mouse IL-6 and determined their apoptosis. Treatment with IL-6 enhanced the apoptotic potential of IL-6^−/−^ fibroblasts following IAV infection in a dose-dependent manner (Fig. 3h) with concomitant decreases in TGF-β production (Fig. 3i). Taken together, lacking of IL-6 rendered lung fibroblasts more resistant to virus-induced apoptosis. Thus, IL-6 may be indispensable for reducing fibroblast proliferation, migration and survival through decreasing TGF-β production.

### IL-6 protects lung epithelial cells from influenza virus-induced apoptosis

**Figure 4:**
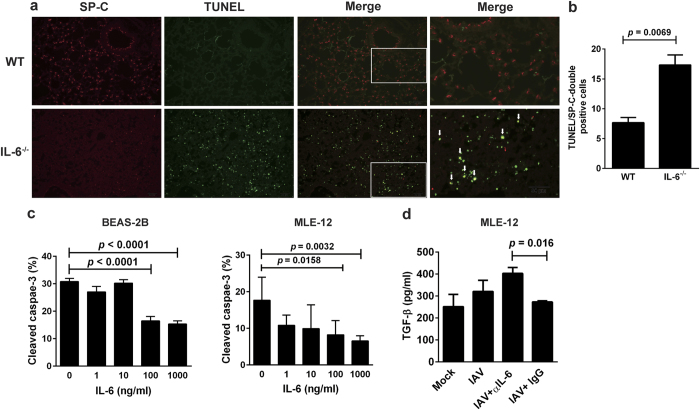
IL-6 deficiency increases influenza virus-induced apoptosis of lung epithelial cells. (**a**) Double staining of lung sections with TUNEL and anti-SP-C antibody (original magnification ×100, scale bar = 50 μm). WT and IL-6^−/−^ mice were infected with 10^5^ PFU of IAV and their lungs were collected at day 7 p.i. for immunohistochemical examination. Arrows indicate TUNEL/SP-C-double positive cells. The boxed areas in the merged panels are magnified and shown in the rightmost panels. (**b**) TUNEL/SP-C-double positive cells were quantified by averaging the number of doubly stained cells in three randomly selected fields in each section (*n* = 3). (**c**) Percentages of apoptotic BEAS-2B (left) and MLE-12 (right) epithelial cells infected with IAV at an MOI of 2 in the presence of different concentrations of IL-6 for 24 h. Apoptosis was determined by immunofluorescence staining with anti-cleaved caspase-3 antibody and quantified by the Celigo cytometry (*n* = 5). (**d**) Levels of TGF-β in the medium collected from MLE-12 cells infected with IAV at an MOI of 2 in the presence of anti-IL-6 neutralizing antibody (αIL-6) or isotype-matched control IgG for 24 h (*n* = 3).

Inappropriate activation of epithelial cells and neutrophil apoptosis can lead to tissue injury and diseases, such as ALI and ARDS^24^. As alveolar type II (AT2) cells can differentiate into alveolar type I (AT1) cells for regeneration of damaged lung tissue, AT2 cells are crucial for reducing epithelial cell apoptosis and promoting lung repair. We thus examined whether IL-6 could protect epithelial cells from IAV-induced death. Lung sections obtained from IL-6^−/−^ and WT mice at day 7 p.i. were double-stained with antibody against the AT2 cell marker surfactant protein C (SP-C) (red) and TUNEL (green). TUNEL-SP-C doubly stained cells were evident in the IL-6^−/−^ lungs, whereas AT2 cells undergoing apoptosis were hardly detectable in the WT lungs (Fig. 4a). Moreover, the numbers of double-positive cells were approximately doubled in the lungs of IL-6^−/−^ mice compared with those of WT mice (Fig. 4b). We further used human bronchial epithelial cells (BEAS-2B) and mouse lung epithelial cells (MLE-12) to assess whether IL-6 reduced IAV-induced epithelial cell apoptosis. Addition of IL-6 decreased the percentage of cleaved caspase-3 in IAV-infected BEAS-2B and MLE-12 cells (Fig. 4c) in a dose-dependent manner. Blocking of IL-6 activity with anti-IL-6 neutralizing antibody in MLE-12 cells infected with IAV increased TGF-β production in the culture medium (Fig. 4d). Taken together, these results indicate that in addition to promoting fibroblast apoptosis, IL-6 improves epithelial cell survival with concomitant inhibition of TGF-β production.

### IL-6 is indispensable for macrophage migration and recruitment to the lung during influenza infection

To determine whether IL-6 played a role in macrophage generation and function, we first examined the differentiation potential of bone marrow cells of WT and IL-6^−/−^ mice into macrophages. Bone marrow cells from both strains of mice were isolated and treated with macrophage colony-stimulating factor (M-CSF) for 7 days to generate bone marrow-derived macrophages (BMDMs). We found that numbers of BMDMs obtained from IL-6^−/−^ and WT mice were not significantly different (Supplementary Figure S2). We also examined whether BMDMs were infectable with IAV. Only less than 4% of BMDMs expressed viral NP, indicative of productive infection with IAV, at 48 h p.i. in either WT or IL-6^−/−^ mice, suggesting low infectability of mouse BMDMs with IAV (Supplementary Figure S3).

**Figure 5:**
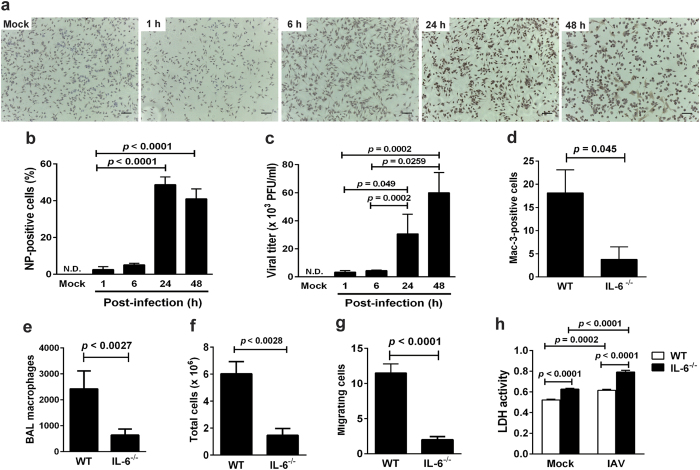
IL-6 deficiency hampers macrophage recruitment to the lung and increases virus-induced cell death during influenza infection. (**a**) Immunohistochemical examination of NP-positive cells in thioglycollate-elicited macrophages infected with IAV at an MOI of 1 for 24 h. Representative images are shown (original magnification ×100, scale bar = 200 μm). (**b**) NP-positive macrophages were quantified by averaging the number of positively stained cells in four randomly selected fields (*n* = 3). (**c**) Titers of IAV produced from the infected macrophages were quantified by the plaque assay (*n* = 3). (**d**) Immunohistochemical detection of macrophages in the lung with anti-Mac-3 antibody at day 7 p.i. in WT and IL-6^−/−^ mice infected with 10^5^ PFU of IAV. Macrophages in the lung were quantified by averaging the number of Mac-3-positive cells in three randomly selected fields in each section (*n* = 4). (**e**) Immunohistochemical detection of macrophages in the BAL fluid with anti-F4/80 antibody at day 7 p.i. in WT and IL-6^−/−^ mice infected with 10^5^ PFU of IAV. Total numbers of macrophages in the BAL fluid were quantified by counting F4/80-positive cells (*n* = 4). (**f**). Total numbers of thioglycollate-elicited peritoneal macrophages collected from WT and IL-6^−/−^ mice were counted using a hemocytometer (*n* = 3 for WT, *n* = 5 for IL-6^−/−^). (**g**) Migratory capability of thioglycollate-elicited peritoneal macrophages from WT and IL-6^−/−^ mice determined by the Boyden chamber assay. The number of migrating cells was the average of the cells counted from four randomly selected fields in each section (*n* = 6). (**h**) Cytotoxicity of thioglycollate-elicited peritoneal macrophages from WT and IL-6^−/−^ mice infected with IAV at an MOI of 1 for 24 h, as assessed by the LDH release assay (*n* = 4).

We next examined whether thioglycollate-elicited peritoneal macrophages, which are convenient sources for mouse macrophages, were permissive for productive infection with IAV. Viral NP was clearly detectable in IAV-infected peritoneal macrophages at 24 and 48 h p.i. by immunohistochemical examination (Fig. 5a). Notably, about 40–50% of the macrophages became productively infected with IAV at 24 h or 48 h p.i. (Fig. 5b), producing 3 × 10^4^–6 × 10^4^ plaque-forming units (PFU)/ml of virus particles (Fig. 5c). These results identified productive replication of IAV in mouse peritoneal macrophages.

Following IAV infection, infiltration of macrophages in the lung (Fig. 5d) and BAL fluid (Fig. 5e) was markedly decreased in IL-6^−/−^ mice at day 7 p.i., as detected by staining with antibodies against macrophage antigens Mac3 and F4/80, respectively. Given that peritoneal macrophages were much more susceptible to productive IAV infection than BMDMs, we used peritoneal macrophages for further studies. Notably, numbers of thioglycollate-elicited peritoneal macrophages isolated from IL-6^−/−^ mice were reduced by 75% compared with those from WT mice (Fig. 5f). Furthermore, lack of IL-6 hampered the migratory response of these macrophages toward fetal bovine serum (FBS) that served as the chemoattractant (Fig. 5g). To investigate whether IL-6 provided survival signals to macrophages, peritoneal macrophages were infected with or without IAV for 48 h, and cell death was analyzed by the lactate dehydrogenase (LDH) release assay. Compared with WT macrophages, more cell death occurred in IL-6^−/−^ macrophages in the absence of IAV infection, which were further increased after viral infection (Fig. 5h). Collectively, these results suggest that presence of IL-6 may promote the migration and recruitment of macrophages to the lung and reduce their death during influenza infection.

### Phagocytic activities of macrophages are decreased in IL-6^−/−^ mice

**Figure 6:**
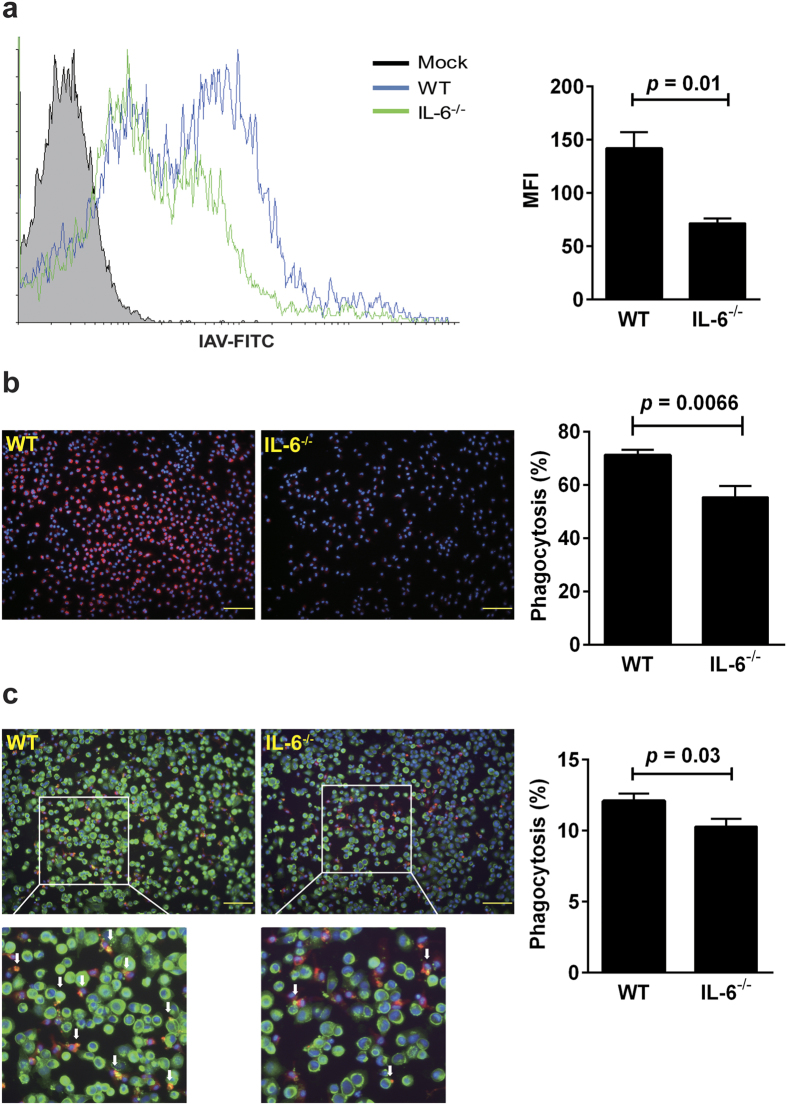
IL-6 deficiency reduces phagocytic activities of macrophages. The phagocytic activities of elicited macrophages collected from WT and IL-6^−/−^ mice were assessed for their uptakes of FITC-labeled IAV (**a**), QD649 quantum dot particles (**b**) and IAV-infected MDCK cells (**c**). (**a**) Phagocytosis of FITC-labeled IAV was measured by flow cytometry and expressed as mean fluorescent intensity (MFI) (*n* = 3). (**b**) The macrophages treated with 5 × 10^10^ QD649 particles for 30 min were stained with DAPI, and their phagocytic activity was determined by the Celigo cytometer. Representative images are shown (original magnification ×200, scale bar = 100 μm). The percentage of phagocytosis was identified as the proportion of macrophages (blue) engulfing QD649 (red) (*n* = 4). (**c**) The macrophages were cultured with biotin labeled-MDCK cells that had been infected with IAV at an MOI of 1 for 2 h, permeabilized, and then fixed for immunofluorescence staining. The cell mixtures were stained with anti-F4/80 antibody and Dylight-488 for detection of macrophages and biotin-labeled MDCK cells, respectively. The phagocytic activity was measured with the Celigo cytometer. Representative images are shown (original magnification ×200, scale bar = 100 μm). The percentage of phagocytosis was identified as the proportion of macrophages (red) engulfing MDCK cells (green) (*n* = 4). Arrows indicate macrophages that engulfed virus infected-MDCK cells (yellow).

Clearance of apoptotic epithelial cells and neutrophils leads to resolution of inflammation and repair^25^. In the infection process, macrophage infiltration as well as ingestion of particles and dead cells by macrophages are important for pathogen clearance and removal of dead cells. To investigate whether IL-6 impacted macrophage phagocytosis, we examined phagocytosis of viral particles and dead infected cells by macrophages from IL-6^−/−^ and WT mice. Flow cytometric analysis shows that FITC-labeled IAV particles were more efficiently ingested by the peritoneal macrophages isolated from WT mice than from IL-6^−/−^ mice (Fig. 6a). Furthermore, similar results were observed when QD649 quantum dots were used for assessing the phagocytic activity of macrophages (Fig. 6b). To mimic real virus-induced cell death, we infected MDCK cells with IAV for 24 h, and then mixed these infected cells with macrophages from IL-6^−/−^ and WT mice at a ratio of 2 : 1 for 2 h. Figure 6c shows that more virus-infected cells were engulfed by WT macrophages than by IL-6^−/−^ macrophages, as observed by fluorescence microscopy and quantified by the Celigo cytometer. Taken together, these results suggest that deficiency in IL-6 may impair phagocytic clearance of influenza virus and virus-infected cells by macrophages.

## Disscusion

Tissue remodeling is crucial for lung repair and regeneration after influenza-induced tissue injury. In the present study, we demonstrate that IL-6 plays a critical role in promoting lung repair in mice with influenza infection through participating in the interplay of macrophages, fibroblasts and lung epithelial cells, as well as through inhibiting TGF-β production.

When normal tissue is damaged, tissue regeneration contains several complicated steps, including inflammation, proliferation and remodeling. Fibroblasts are involved in the proliferation and remodeling phases. The roles of TGF-β in the interplay between fibroblasts and epithelial cells have been studied in much detail. TGF-β has a contrast role in epithelial cells. It induces apoptosis in bronchiolar epithelial cells and diminishes lung epithelial regeneration^26^. Moreover, TGF-β promotes epithelial cells undergoing EMT to become myofibroblasts^27^.Targeting TGF-β activity and its downstream signaling pathways effectively attenuates fibrosis formation^28^,^29^. Previous studies have indicated that IL-6 and TGF-β participate in the pathogenesis of lung diseases, such as ARDS^30^, lung fibrosis, asthma^31^ and chronic obstructive pulmonary disease^32^. Influenza virus induces activation of latent TGF-β, which can determine viral pathogenesis and is associated with virus-induced cell apoptosis^4^,^12^. Influenza virus-infected mice with heterozygous mutation in the cystic fibrosis transmembrane conductance regulator had higher levels of IL-6 and alveolar macrophages in the BAL fluid, and did not develop ALI^33^. Such effects were associated to TGF-β-dependent production of IL-6^33^. Recently, it was shown that integrin β6 subunit gene knockout mice had increased survival after influenza infection and reduced ALI, which were attributed to the loss of β6-activated TGF-β and increases in activated CD11b^+^ alveolar macrophages and type I interferon signaling in the lung^34^. These results are in accordance with our findings. We show that IL-6 is essential for the survival and the recovery from severe lung injury in mice after influenza infection, which is associated with reduced TGF-β production. Loss of IL-6 interferes with the functions of lung fibroblasts, including increases in proliferation rate and migratory capability, as well as resistance to virus-induced apoptosis, which may be mediated by increased TGF-β production. Notably, accumulation of fibroblasts in the lung causes deposition of collagen and fibronectin. Furthermore, addition of recombinant IL-6 to the lung epithelial cells infected with influenza virus decreased caspase-3 activation, indicative of enhanced survival, and reduced TGF-β production. Collectively, we identify a novel role for IL-6 in the lung repair process.

Depletion of alveolar macrophages, which are critical for host defense against influenza, leads to increased susceptibility to influenza virus infection and massive pathology in pigs and mice^35^,^36^. Effects of IL-6 on immune cells have been extensively studied. IL-6 controls monocyte differentiation into macrophages^37^. Migration and infiltration of macrophage are also dependent on the IL-6 and Stat3 signaling pathway^38^. A recent report has shown that bone-marrow derived dendritic cells from IL-6-deficient mice displayed defects of phagocytosis of fluorescent carboxylate-modified polystyrene latex beads^31^. These findings are in agreement with our results that IL-6 is essential for alleviating influenza symptoms and subsequent lung injury by promoting macrophage recruitment to the lung and by phagocytosing virus-infected cells. Whether these activities of macrophages serve to enhance virus clearance or reduce lung inflammation is currently not clear, but warrants further investigation.

Several lines of evidence have suggested that TGF-β and IL-6 can regulate each other in different circumstances. IL-6 increases trafficking of TGF-β receptor to non-lipid raft-associated pools, resulting in augmented TGF-β1/Smad signaling^39^. Moreover, TGF-β1-induced IL-6 expression participates in trans-differentiation of fibroblasts to myofibroblasts^40^. By contrast, TGF-β inhibits IL-6 signaling by reducing Stat activity in the intestinal epithelial cells, and serves as a negative regulator in uncontrolled inflammation^41^. We show that TGF-β production is upregulated in the lung of IL-6 knockout mice. Furthermore, addition of recombinant IL-6 to IL-6^−/−^ fibroblasts reduces virus-induced TGF-β production, whereas addition of neutralizing IL-6 antibody in lung epithelial cells increases TGF-β production. Our results are partially consistent with a previous study showing that hepatocyte growth factor and IL-6 inhibit TGF-β-mediated fibroblast-myofibroblast transition through reduction of α-SMA expression^42^. These findings suggest that IL-6 and TGF-β may positively or negatively regulate each other under different conditions.

Not only does IL-6 promote host defense to pathogen invasion, it also resolves disease onset. We identify a novel role for IL-6 produced by fibroblasts during influenza infection in promoting apoptosis and reducing proliferation of fibroblasts, as well as balancing fibroblast migration through downregulating TGF-β production. Moreover, IL-6 is essential for macrophages to phagocytose virus-infected cells. In influenza infection, mice deficient in IL-6 had decreased survival and more severe lung injury. Therefore, we demonstrate that IL-6 not only acts as an immune regulator for defending against influenza, but also plays an important role in balancing lung environment.

## Methods

### Mice, cells and viruses

Female C57BL/6 mice were purchased from the Laboratory Animal Center of National Cheng Kung University (NCKU) or National Laboratory Animal Center (Taipei, Taiwan). IL-6^−/−^ (B6.129S2-*Il6*^tm1kopf^/J) mice with C57BL/6 background were purchased from Jackson Laboratory and maintained in the Laboratory Animal Center of NCKU. MDCK cells were cultured in Dulbecco’s modified Eagle’s medium (DMEM) supplemented with 10% cosmic calf serum (Hyclone, Logan, UT), 2 mM L-glutamine and 50 μg/ml gentamicin. Primary lung fibroblasts were isolated from WT and IL-6^−/−^ mice, maintained in DMEM with 10% cosmic calf serum, 2 mM L-glutamine and 50 μg/ml gentamicin, and used between the fourth and seventh passages. Human bronchial BEAS-2B epithelial cells were maintained in BEBM medium (Lonza, Rockland, ME). Mouse MLE-12 epithelial cells were maintained in F12 medium with 4% FBS, 2 mM L-glutamine, 0.1 mM non-essential amino acid, 50 μg/ml gentamicin, 5 μg/ml insulin, 10 ng/ml epidermal growth factor, 1 μg/ml transferrin and 500 ng/ml hydrocortisone. Influenza A/WSN/33 (H1N1) virus was propagated and titrated in MDCK cells as described previously^43^. All *in vitro* work on influenza virus was carried out in biosafety level 2 laboratories. All animal work was conducted in animal biosafety level 2 facilities at NCKU. The experimental protocols adhered to the rules of the Animal Protection Act of Taiwan and were approved by the Animal Care and Use Committee of NCKU (IACUC number: 104088).

### Animal studies

Groups of female C57BL/6 mice and IL-6^−/−^ mice at 4–6 weeks of age were intranasally inoculated with 10^5^ PFU of IAV which corresponded to 1.5 × lethal dose (LD_50_) at day 0. The mice were monitored daily for illness, weight loss and death for 16 days after viral infection.

### Histochemical, immunohistochemical, immunofluorescence and apoptotic analyses

IAV-infected mice that had received different treatments were killed at day 7 or 10 p.i. The lungs were removed, formalin-fixed and paraffin-embedded for hematoxylin and eosin (H&E) staining using standard methods. Inflammatory changes on the basis of numbers of inflammatory cells and tissue damage in the lungs were determined by histology from H&E-stained longitudinal cross sections and scored on a 0–3 scale (0 = no change, 1 = mild, 2 = moderate, 3 = severe)^43^. For immunohistochemical staining, tissue sections were deparaffinized, antigen-retrieved using protease K (100 μg/ml, Life Technologies, Carlsbad, CA) digestion for 10 min at room temperature and incubated with rabbit anti-human SFP-1 (S100A4) antibody (1:400, Abcam, Cambridge, UK), rabbit anti-human fibronectin antibody (1:200, Santa Cruz Biotechnology, Santa Cruz, CA), rat anti-mouse Mac3 antibody (1:50; M3/84, BD Biosciences PharMingen, San Diego, CA) and monoclonal mouse anti-α-SMA antibody (1:400, Sigma-Aldrich, St. Louis, MO). After sequential incubation with appropriate horseradish peroxidase (HRP)-conjugated secondary antibody at room temperature and 3-amino-9-ethyl carbazole (AEC) as the substrate chromogen, the slides were counterstained with hematoxylin. The signal intensity of immunohistochemical staining was further quantified using the Image J software (https://imagej.nih.gov/ij/). To detect apoptotic epithelial cells in the lungs, paraffin-embedded lung tissue sections were subjected to co-stain with goat anti-mouse SP-C (M-20) antibody (1:200, Santa Cruz) and TUNEL according to the manufacturer’s instructions (Promega, Madison, WI). Lung fibroblasts, BMDMs and peritoneal macrophages from WT and IL-6^−/−^ mice, as well as BEAS-2B and MLE-12 cells were used for viral infection and apoptosis assay. These cells were infected with IAV at a multiplicity of infection (MOI) of 1 (for fibroblasts and macrophages) or 2 (for BEAS-2B and MLE-12 cells) for 24 h and fixed with 4% formaldehyde. Virus-infected cells were detected by mouse monoclonal anti-IAV NP antibody (1:1000, Abcam) and goat anti-mouse IgG (1:200, Life Technologies). Apoptotic cells were examined by rabbit monoclonal anti-human cleaved caspase-3 antibody (1:1000, Cell Signaling Technology, Danvers, MA) and Alexa Flour 488-labeled goat anti-rabbit IgG (1:200, Life Technologies). The numbers or percentages of positively stained cells were calculated by the Celigo cytometer or observed under fluorescence microscopy.

### ELISA

BAL was performed as described previously^43^. Lung fibroblasts collected from WT and IL-6^−/−^ mice or MLE-12 were infected with IAV at MOI of 1 and 2, respectively, in the presence of rabbit anti-IL-6 neutralizing antibody (1:400, Abcam) or isotype-matched control IgG for 24 h. The levels of TGF-β and IL-6 cytokines in the BAL fluid and culture medium were quantified using DuoSet ELISA kits (R&D, Minneapolis, MN).

### Fibrosis and edema

Lung sections collected from mice infected with IAV at days 15 and 28 p.i. were stained with picrosirius red to determine the degree of collagen deposition. To assess lung edema, the wet/dry ratio of the infected lungs was assessed at day 7 p.i. The lungs were dissected, weighed, and dried at 60 °C for 2 days. The wet/dry ratio was then calculated by dividing the wet weight by the final dry weight.

### Fibroblast functional assay

To assess fibroblast functions, we analyzed the proliferation and migration capabilities of lung fibroblasts collected from WT and IL-6^−/−^ mice. Fibroblasts were cultured in DMEM containing 10% FBS for 24, 48 and 72 h. The proliferation rate and doubling time were calculated by the Celigo cytometer. Migratory capabilities of fibroblasts were analyzed using the Boyden chamber assay. The cells were placed in the upper compartment and allowed to migrate through the pores of the membrane into the lower compartment, in which the conditioned medium from each cell type after infection with IAV for 24 h served as the chemoattractant, and incubated for 24 h. The migrated cells were fixed by methanol and stained with Giemsa. The number of migrating cells was the average of the cells counted in three randomly selected fields in each well.

### Macrophage functional assays

Macrophages collected from WT and IL-6^−/−^ mice after peritoneal injection with thioglycollate (3%) for 3 days were used to analyze the migratory capability of macrophages by the Boyden chamber assay. The macrophages were placed in the upper compartment and allowed to migrate through the pores of the membrane into the lower compartment, in which FBS served as the chemoattractant, and incubated for 24 h. The migrating cells were fixed by methanol and stained with Giemsa. The number of migrating cells was the average of the cells counted in three randomly selected fields in each section. To assess the phagocytosis of viruses or nanoparticles by macrophages, peritoneal macrophages (10^6^ cells) were incubated with IAV that had been labeled with FITC (NHS-Fluorescein; Pierce, Rockford, Ill) at an MOI of 1 as described previously^44^ or with 5 × 10^10^ QD649 quantum dot particles for 30 min at 37 °C and then treated with 40 μl of 0.1% trypan blue to quench extracellular florescence. Macrophages were then stained with DAPI. After being washed with phosphate-buffered saline (PBS), florescence was analyzed by flow cytometry (BD Biosciences, San Diego, CA) or the Celigo cytometer, and photographed with fluorescence microscopy. Furthermore, the phagocytosis of virus-infected cells by macrophages was also assessed. MDCK cells that had been infected with IAV at an MOI of 1 and then labeled with biotin (NHS-LS-Biotin; Pierce) were mixed with macrophages (at a ratio of two virus-infected cells to one macrophage), and incubated at 37 °C for 2 h. The cell mixture was washed with PBS, fixed with 3.7% formaldehyde, permeabilized with 0.01% Triton X-100 and then added with Dylight488-conjugated streptavidin (1:200, Jackson ImmunoResearch, West Grove, PA). Macrophages were detected by rat anti-mouse F4/80 antibody (1:50, Serotec, Oxford, UK). The number of macrophages containing engulfed cells was determined using fluorescence microscopy and the Celigo cytometer. The percentage of phagocytosis was calculated as the number of engulfing macrophages relative to the total number of macrophages. For the survival assay, macrophages (10^4^ cells) were infected with IAV for 48 h, and the cytotoxicity was measured by CytoTox 96 non-radioactive cytotoxicity assay (Promega).

### Statistical analysis

Data are expressed as mean ± standard deviation (SD). Differences in body weights between two groups were compared by repeated-measures analysis of variance (ANOVA). The survival analysis was performed using the Kaplan-Meier survival curve and log-rank test. For the remaining data, statistical differences were compared by Student’s *t*-test between two groups and by one-way ANOVA with Bonferroni post hoc test among three or more groups. The differences were considered significant if *P* values were < 0.05. Statistical tests were performed using GraphPad Prism (version 6.0, GraphPad software, San Diego, CA).

## Additional Information

**How to cite this article:** Yang, M.-L. *et al*. IL-6 ameliorates acute lung injury in influenza virus infection. *Sci. Rep.* **7**, 43829; doi: 10.1038/srep43829 (2017).

**Publisher's note:** Springer Nature remains neutral with regard to jurisdictional claims in published maps and institutional affiliations.

## Supplementary Material

Supplementary Information
