# Supplementary material for: IL-6 ameliorates acute lung injury in influenza virus infection
Source: Sci Rep. 2017 Mar 6;7:43829. doi: 10.1038/srep43829 (PMC5338329; doi:10.1038/srep43829)
Supplement: Supplementary Information [file srep43829-s1.pdf]

IL-6 ameliorates acute lung injury in influenza virus infection

Mei-Lin Yang[1], Chung-Teng Wang[1], Shiu-Ju Yang[1], Chia-Hsing Leu[2], Shun-Hua Chen[1], Chao-Liang Wu[2*], Ai-Li Shiau[1*]

[1]Department of Microbiology and Immunology, College of Medicine, National Cheng Kung University, Tainan, Taiwan

[2]Department of Biochemistry and Molecular Biology, College of Medicine, National Cheng Kung University, Tainan, Taiwan

[*]Correspondence to: Ai-Li Shiau, Department of Microbiology and Immunology, College of Medicine, National Cheng Kung University, 1 University Road, Tainan 70101, Taiwan. Phone: +886-6-2353535 ext. 5629. Fax: +886-6-2082705. E-mail: alshiau@mail.ncku.edu.tw; or Chao-Liang Wu, Department of Biochemistry and Molecular Biology, College of Medicine, National Cheng Kung University, 1 University Road, Tainan 70101, Taiwan. Phone: +886-6-2353535 ext. 5536. Fax: +886-6-2741694. E-mail: wumolbio@mail.ncku.edu.tw.

SUPPLEMENTARY MATERIALS

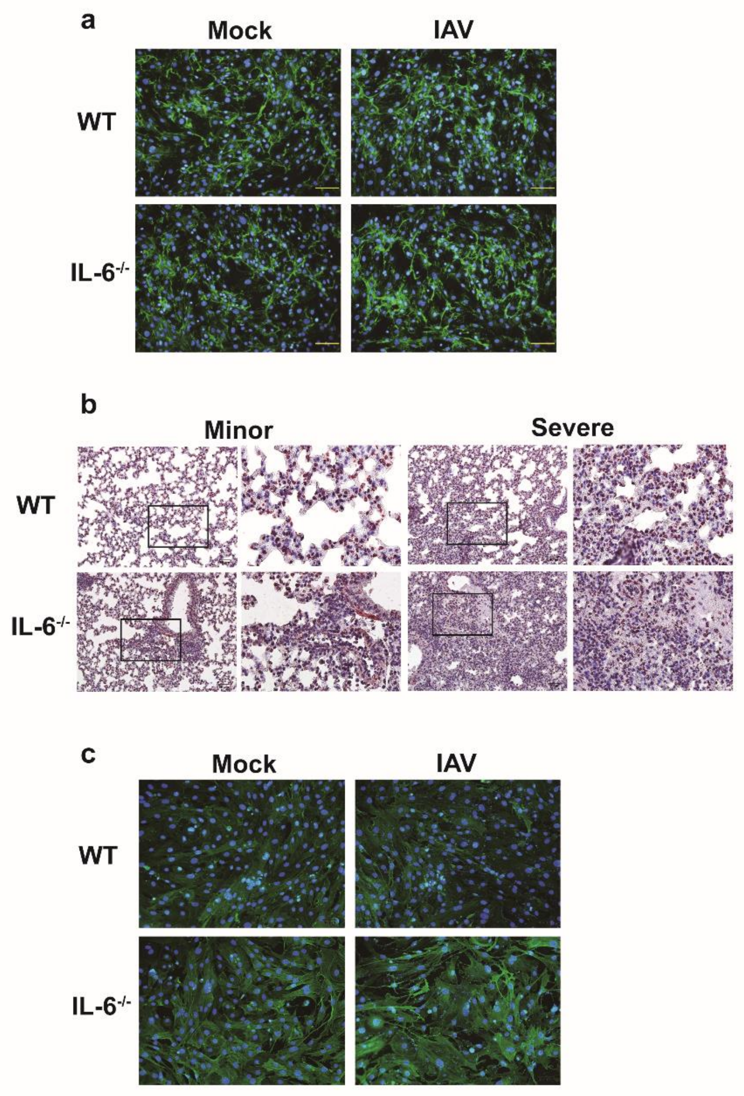

Supplementary Figure S1. Deficiency in IL-6 does not affect epithelial-mesenchymal transition (EMT). (a) WT and IL-$6^{-/-}$ fibroblasts were infected with IAV at an MOI of 1 for 24 h, and then stained with anti-fibronectin antibody, followed by FITC-conjugated secondary antibody (original magnification ×200, scale bar = 100 μm). (b) WT and IL-$6^{-/-}$ mice were intranasally inoculated with IAV (10^5 PFU). Paraffin-embedded lung sections were prepared at day 7 p.i. for staining with anti-α-SMA antibody, followed by HRP-conjugated secondary antibody and AEC as the substrate chromogen (original magnification ×200, scale bar = 50 μm). Representative images from lung sections exhibiting minor or severe fibroblast accumulation. (c) WT and IL-$6^{-/-}$ fibroblasts were infected with IAV at an MOI of 1 for 24 h, and then stained with anti-α-SMA antibody, followed by FITC-conjugated secondary antibody (original magnification ×200, scale bar = 50 μm).

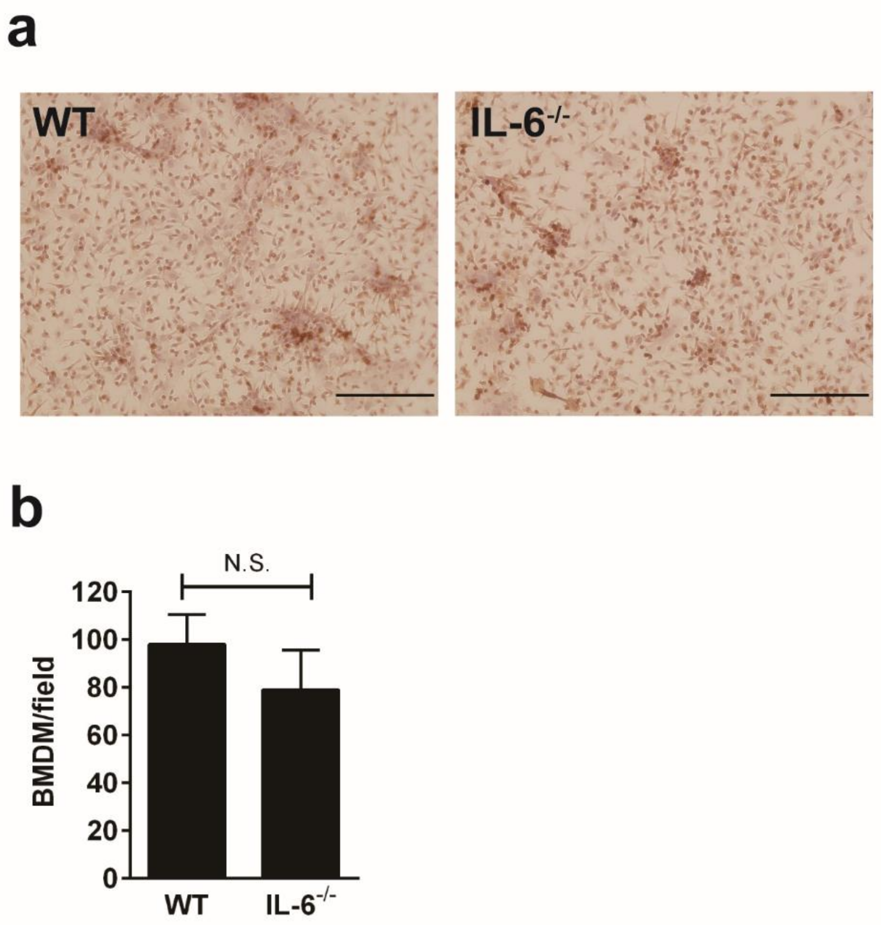

Supplementary Figure S2. Deficiency in IL-6 does not affect differentiation of bone marrow cells to macrophages. (a) Bone marrow cells isolated from WT and IL-$6^{-/-}$ mice were treated with M-CSF for 7 days to generate bone marrow-derived macrophages (BMDMs). They were then stained with anti-F4/80 antibody, followed by HRP-conjugated secondary antibody and AEC as the substrate chromogen. Representative images are shown (original magnification × 400, scale bar = 100 μm). (b) Positively stained cells were quantified by averaging the number of stained cells in four randomly selected fields. Values shown are the mean ± SD (n = 4). N.S., non-significant.

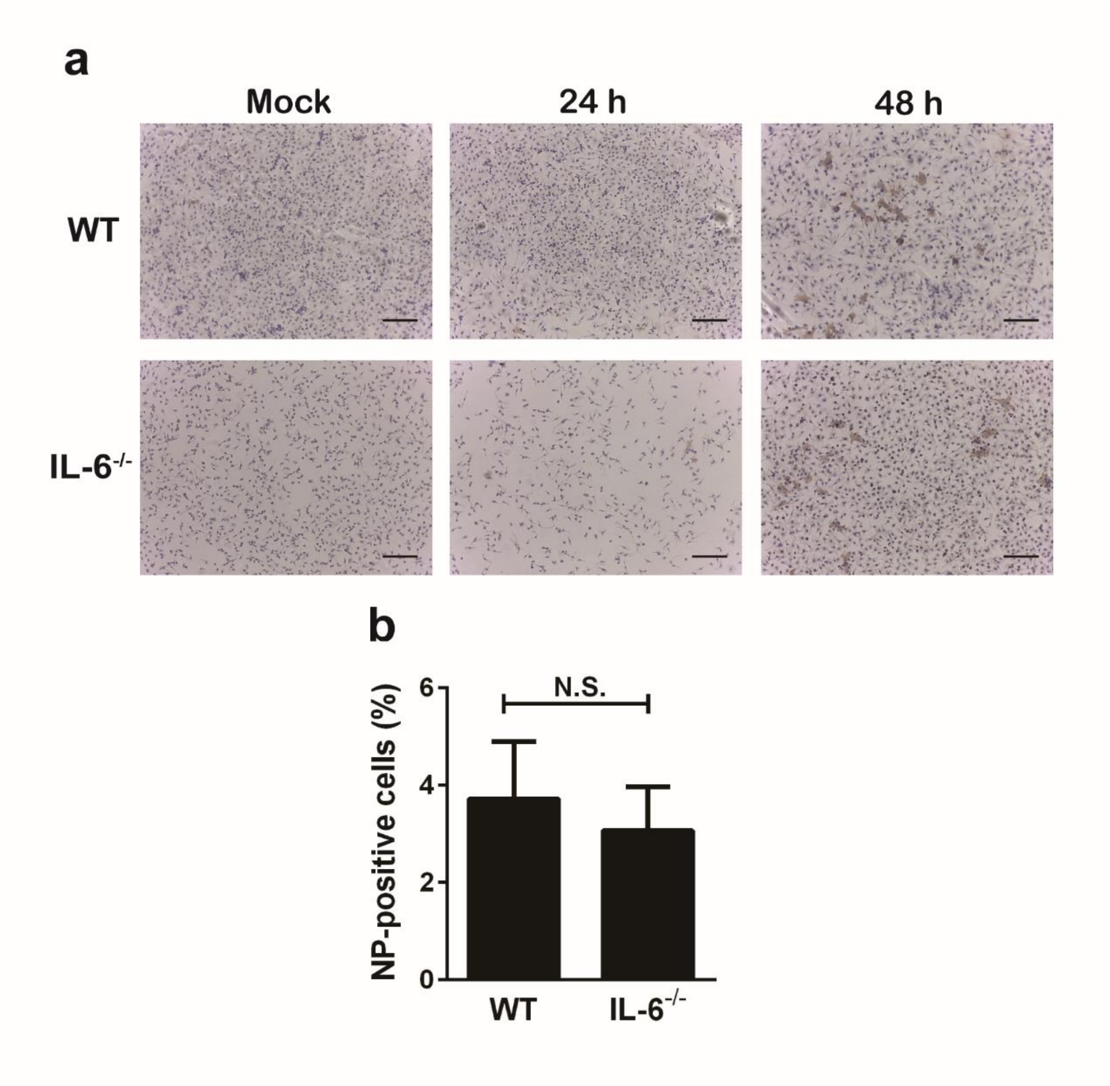

Supplementary Figure S3. BMDMs of IL-6$^{-/-}$ and WT mice exhibit low infectability with IAV. (a) BMDMs were infected with IAV at an MOI of 1 or mock-infected for 24 h or 48 h, and then fixed. Virus-infected cells were stained with anti-IAV NP antibody, followed by HRP-conjugated secondary antibody and AEC as the substrate chromogen. Representative images are shown (original magnification × 100, scale bar = 200 μm). (b) Positively stained cells at 48 h p.i. were quantified by averaging the number of stained cells in four randomly selected fields. Values shown are the mean ± SD (*n* = 3). N.S., non-significant.
